# Experimental piscine alphavirus RNA recombination *in vivo* yields both viable virus and defective viral RNA

**DOI:** 10.1038/srep36317

**Published:** 2016-11-02

**Authors:** Elin Petterson, Tz-Chun Guo, Øystein Evensen, Aase B. Mikalsen

**Affiliations:** 1 Norwegian University of Life Sciences, Faculty of Veterinary Medicine and Biosciences, P.O. Box 8146 Dep., N-0033 Oslo, Norway

## Abstract

RNA recombination in non-segmented RNA viruses is important for viral evolution and documented for several virus species through *in vitro* studies. Here we confirm viral RNA recombination *in vivo* using an alphavirus, the SAV3 subtype of Salmon pancreas disease virus. The virus causes pancreas disease in Atlantic salmon and heavy losses in European salmonid aquaculture. Atlantic salmon were injected with a SAV3 6K-gene deleted cDNA plasmid, encoding a non-viable variant of SAV3, together with a helper cDNA plasmid encoding structural proteins and 6K only. Later, SAV3-specific RNA was detected and recombination of viral RNA was confirmed. Virus was grown from plasmid-injected fish and shown to infect and cause pathology in salmon. Subsequent cloning of PCR products confirming recombination, documented imprecise homologous recombination creating RNA deletion variants in fish injected with cDNA plasmid, corresponding with deletion variants previously found in SAV3 from the field. This is the first experimental documentation of alphavirus RNA recombination in an animal model and provides new insight into the production of defective virus RNA.

Alphavirus is one of two genera in the family *Togaviridae*^1^. They are small, enveloped viruses that infect a broad range of insect and vertebrate hosts. Currently there are 31 assigned virus species within the genus (International Committee on Taxonomy of Viruses, 2015), where two virus species infecting marine animals are identified, southern elephant seal virus (SESV) and salmon pancreas disease virus (SPDV)^2^,^3^. Alphaviruses are classified as arboviruses (arthropod-borne virus) transmitted to vertebrates by hematophagous insects^1^. SPDV infects salmonids and can be transmitted without an insect vector^4^. SPDV, also called salmonid alphavirus (SAV), is divided into subtype 1–6^5^ and causes pancreas disease (PD) and severe disease problems with high economic losses in salmonid aquaculture in Europe. The SPDV genome is an 11.9 kb, positive sense ssRNA molecule, capped (5′) and polyadenylated (3′) and serves directly as mRNA for translation of the viral replicase when it enters a host cell^1^,^6^. As for all alphavirus, the RNA molecule contains two open reading frames (ORFs). The first ORF encodes a polyprotein which is processed into four non-structural proteins (nsP1–4), responsible for transcription and replication of viral RNA. The second ORF is transcribed as a subgenomic mRNA and translated into a polyprotein subsequently processed into the structural proteins (capsid, E3, E2, 6K and E1). The cleavage sites in both polyproteins are deduced from amino acid sequence homologies with other alphaviruses^3^,^6^,^7^,^8^. E2 and E1 are the envelope glycoproteins and it is shown for other alphaviruses that they are associated as a heterodimer that migrates together with the membrane-associated 6K protein to the plasma membrane^9^.

Recombination in RNA viruses refers to the process that occurs within a single genomic segment during virus replication. The copy-choice model describes polymerase switching between templates during RNA synthesis^10^,^11^, and is the most supported model, but other mechanisms and models might also apply. Two types of RNA recombination have been defined, homologous and heterologous, both documented in alphavirus *in vitro*^12^. The first known incidence of alphavirus recombination is the emergence of Western equine encephalitis virus (WEEV), a recombination of an Eastern equine encephalitis (EEEV)-like virus and a Sindbis-like virus^13^, later giving rise to Highlands J virus (HJV), and Fort Morgan virus (FMV)^1^. Recombination of Sindbis virus RNA *in vitro* has been documented experimentally in transfected cells in the early nineties^12^,^14^. RNA recombination may also result in formation of defective interfering (DI) RNAs^15^. Thus RNA virus recombination plays an important role in virus variability and evolution, and can also play a role in rescuing viral genomes by repairing mutation errors introduced during RNA replication^16^. Experimentally, RNA recombination of alphaviruses has not been shown *in vivo* across all susceptible species.

In a previous study on the SAV3 subtype of SPDV, we documented that infectious virus (with full-length genome) is present alongside with numerous RNA deletion mutants in heart of clinically diseased Atlantic salmon, and we hypothesised that recombination of genomes was the explanation for this^17^. Using reverse genetics, we have shown that infectious virus can be rescued from cell lines transfected with full length SAV3 (FL-SAV3) plasmid^18^. When cell cultures were transfected with a full length but 6K-deleted construct (FL-∆6K), viral proteins were expressed in transfected cells, but viable, infectious virus was not formed^18^. When we co-transfected cell cultures with the FL-∆6K plasmid and a helper plasmid encoding the structural proteins including 6K (Helper-6K), infectivity was rescued through viral RNA recombination *in vitro* and formation of infectious virus^18^. This provided us with a tool to explore if the same recombination would occur *in vivo*. We used Atlantic salmon, one of the species susceptible to SAV3 infection, and we have documented for the first time recombination of alphavirus RNA in an animal model. Virus was grown in cell culture from heart of FL-∆6K/Helper-6K injected fish and shown to be pathogenic to salmon in experimental challenge.

## Results

### Presence of viral RNA transcripts in plasmid-injected fish

**Figure 1:**
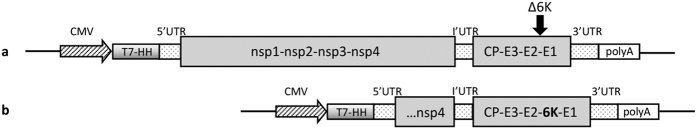
Organization map of SAV3 cDNA in plasmids. (**a**) SAV3 6K-deleted cDNA plasmid (FL-∆6K): the entire 6K gene was removed from SAV3 full-length genome. (**b**) Helper cDNA plasmid (Helper-6K): 100 bases of nsp4 C-terminal together with the whole 26S subgenome containing 6K gene were included in the helper plasmid. HH: hammerhead ribozyme sequence, a self-cleavage ribozyme providing the exact 5′ viral end after RNA transcription from cDNA plasmid.

**Figure 2:**
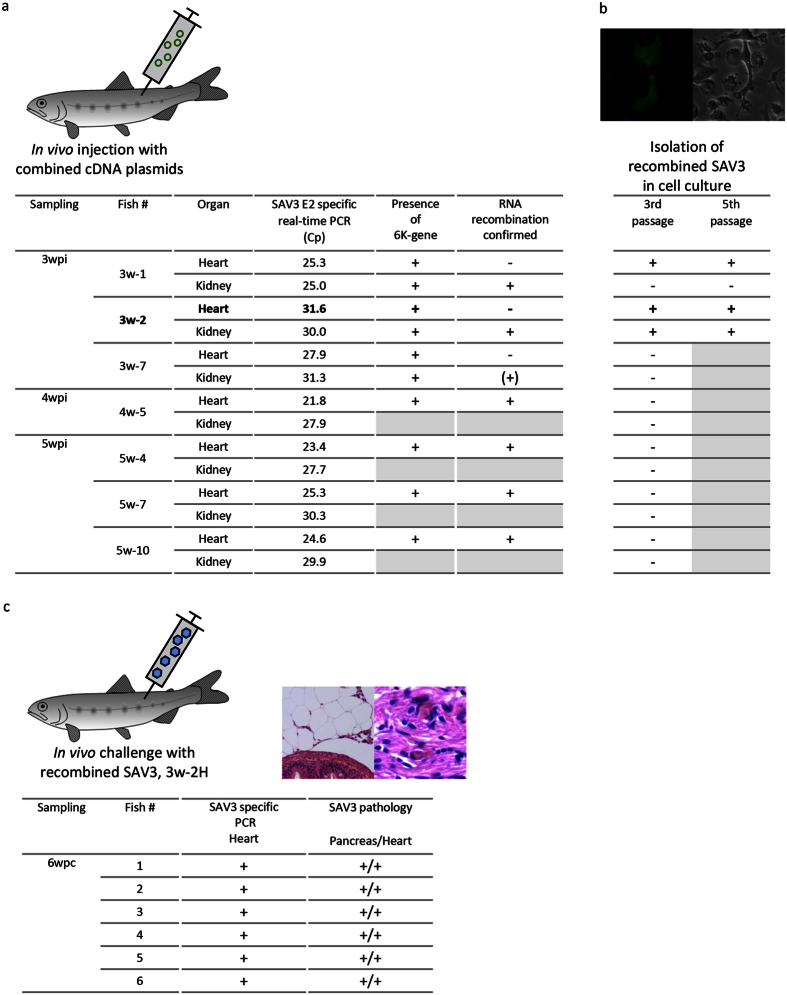
Experimental overview and main results. (**a**) Atlantic salmon injected with a combination of SAV3 FL-Δ6K and Helper-6K cDNA plasmids. Results show fish with presence of SAV3 RNA in heart and kidney tissue as Cp-values (individuals with no presence are not shown) at the specified sampling time (weeks post injection (wpi)) and as presence of 6K-gene found by PCR (+). The outcome of PCR analysis for RNA recombination in heart and kidney tissue is shown, and + denotes presence of an amplicon with unique sequences from SAV3-FL-∆6K and Helper-6K plasmids, confirmed by sequencing. Four to seven plasmid clones were sequenced from each PCR-positive fish, with the exception of fish 3w-2 where a very low concentration of the 2.9kb product only resulted in one clone. All clones include the sequence unique for the SAV3-FL-∆6K plasmid and at least one clone per fish also includes 6K or incomplete 6K sequences unique for the Helper-6K. No clones show a sequence equal to any of the two plasmids alone. Brackets indicate presence of amplicon where sequences were not obtained; −denotes no amplicon present. (**b**) Primary isolation of recombinant SAV3 in CHH-1 cell culture. Left image shows example of positive staining for SAV3 spike proteins in 2^nd^ passage of cells inoculated with heart tissue from fish 3w-1 or 3w-2. CPE was not seen in this passage. Right image show example of CPE, 5^th^ passage. CPE (+) in cell cultures inoculated with tissue from the fish positive for SAV3 RNA. Results for 3^rd^ and 5^th^ passage shown. −denotes no CPE. (**c**) Atlantic salmon injected with SAV3 from the supernatant of the 5^th^ passage (isolate 3w-2H, i.e. originating from heart tissue of fish 3w-2, shown in bold in (**a**,**b**). Results show fish with presence (+) of SAV3 RNA in heart tissue and evidence of pathology characteristic of SAV3 infection in pancreas and heart by light microscopy. Image show loss of exocrine pancreas located in perivisceral fat tissue (left) and necrotic myocytes in spongiosum of the heart ventricle (right). Grey shading indicates no analyses performed.

First, we aimed to document presence of viral RNA transcripts following injection with plasmid/plasmid mixtures. Atlantic salmon parr were injected intramuscularly (i.m.) with a combination of a cDNA plasmid expressing full-length SAV3 genome with the 6K-gene deleted (FL-∆6K) and a helper cDNA vector (Helper-6K) encoding the structural genes including 6K (Fig. 1). SAV3-specific RNA, part of the E2 gene, was detected by real-time PCR in heart and head kidney in 3 of 10 individuals at 3 weeks post injection (wpi, Fig. 2a). Thereafter, 1 of 6 individuals was positive for viral RNA at 4 wpi and 3 of 10 fish at 5 wpi, in total 7 PCR-positive fish (Fig. 2a). The amount of viral RNA detected (given as Cp-values) varied between 21.8 and 31.6, with no obvious trends (increase/decrease) over time post injection. The range of viral RNA levels detected (i.e. Cp-values) is in conformity with levels seen in experimental challenges using live virus^19^. Viral RNA was not detectable by PCR at 1 and 2 wpi (not shown). No SAV3-specific RNA was found in any organ at any time point post injection in the group injected with FL-∆6K alone or in negative control fish (not shown). Negative control fish were also negative at start of the experiment.

Experimental infection with live virus results in pathological changes in heart, pancreas and skeletal muscle when given at doses above 10^3^ TCID_50_^20^. Here, no lesions were recorded (by histological examination) in any of the sampled organs. To ascertain that PCR positive signals did not originate from injected plasmids contaminating the RNA, all RNA was DNase-treated at time of isolation. Absences of plasmid DNA contamination was also verified by application of a similar real-time PCR procedure on a selection of parallel RNA aliquots without transcription into cDNA (preceding PCR). No amplification would then be possible from the RNA but DNA plasmid contamination would yield a product. These reactions resulted in no products. These findings indicate transcription of viral genome and/or replication of virus, and the fact that RNA was not detected by PCR in samples during the first two weeks post injection corroborates the same.

### Recombination is confirmed *in vivo*

**Figure 3:**
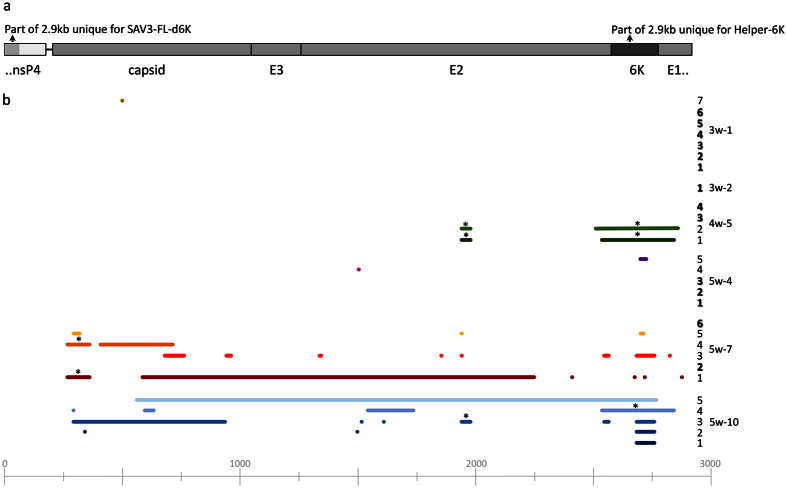
Sequencing of PCR products from recombined virus RNA. (**a**) Overview of full-length 2.9kb PCR product including sequences unique for SAV3 FL-Δ6K and Helper-6K (**b**) Deletions per single PCR product clone relative to full-length 2.9kb product are marked as lines, dots represent deletions of <10 nt. Colour coding has been used to visualize fish individuals and colour shades differentiating each clone, also numbered with digits. Clone numbers in bold is used on clones with no deletions. Asterisks denote deletion similar to earlier published in Petterson *et al*.^17^.

The 6K gene is required for replication and production of infectious virus^18^. To verify presence of 6K in the 7 PCR-positive fish (by real-time PCR, Fig. 2a), we examined these fish using conventional PCR and primers yielding an amplicon covering the 6K gene (380bp) and all were found positive (Fig. 2a), indicating that a replicating virus including 6K gene in the genome could have resulted from recombination. To confirm this, we amplified a larger product, 2.9 kb spanning the end of nsp4 and the structural proteins including 6K into E1 (Fig. 3a). The primers were designed to include a unique 67nt stretch of nsp4 for the SAV3-FL-∆6K plasmid while the entire 6K frame was unique for the Helper-6K (Fig. 3a). A product including both sequences would only be present if recombination had occurred. In all of the 7 individuals positive for the 380bp 6K amplicon, the 2.9kb amplicon was produced to detectable levels. Cloning and sequencing confirmed that sequences, unique for the two plasmids, were included (Fig. 2a), with the exception of fish 3w-7, which resulted in a 2.9 kb amplicon with too low concentration for cloning and sequencing (Fig. 2a). A similar PCR was performed using the primers for the 2.9 kb amplicon on a mix of purified preparations of the DNA plasmids, i.e. FL-∆6K and Helper-6K as template, together with appropriate controls. The mix of the FL-∆6K and Helper-6K gave a 2.7 kb amplicon consistent with amplification from FL-∆6K only, which confirms that the recombination is unlikely to have happened during PCR. Collectively, these results confirm recombination *in vivo*.

### Primary isolation of recombinant SAV3 in CHH-1 cell culture

Our next focus was to show that virus could be isolated from target/internal organs. Organ material (heart/kidney) from the real-time PCR positive fish (Fig. 2a) were processed for inoculation onto permissive cells, CHH-1, with subsequent passaging. No cytopathic effect was evident during the first two passages of any tissue samples tested, but we obtained positive Immunofluo-rescent staining for SAV3 spike proteins of 2^nd^ passage cells inoculated with heart tissue from individuals 3w-1 and 3w-2, 3 days post inoculation (dpi) (Fig. 2b). At 3^rd^ passage, cytopathic effect (CPE) appeared for fish no. 3w-1 and 3w-2 (Fig. 2b). For the latter, CPE was also found for kidney tissue inoculated cells. Early signs of CPE appeared, as vacuoles at 6 dpi and later at 10 dpi swelling and detached single cells were dominant. Subsequent passages showed typical CPE at 4 dpi, progressing to cell lysis at 8–10 dpi (Fig. 2b). Supernatant from 5^th^ passage of heart tissue, fish 3w-2 (isolate 3w-2H), was used for challenge studies. The obtained titre was 10^6.8^ TCID_50_/ml, and before subsequent use RNA including recombined sequences were confirmed as described above (amplification of unique SAV3 FL-∆6K encoded RNA and Helper-6K RNA). The entire viral genome of 3w-2H isolate was also sequenced confirming it to be identical to the original SAV3-H10 genome sequence, with the exception of a C1375G mutation of nsp3, resulting in a Q459E substitution.

### Recombinant SAV3 induces pathology in Atlantic salmon parr

Since we were not able to confirm any pathological changes in the plasmid-injected fish we used isolate 3w-2H (5^th^ passage, Fig. 2b) to challenge Atlantic salmon parr by i.m. injection in a second *in vivo* experiment. SAV3 RNA was detected by PCR in all individuals sampled at 4 and 6 wpi (Fig. 2c). Pathological changes specific for SAV3-infection were found in heart, 4/6 fish at 4 wpi and 6/6 at 6 wpi, and in exocrine pancreas in 6/6 fish at both time points (Fig. 2c). Fish with typical changes in pancreas^21^ (Supplementary Fig. 1a) also showed presence of SAV3 antigens, shown by immunohistochemistry (Supplementary Fig. 1b).

### Imprecise homologous recombination creates RNA deletion variants

One thing that puzzled us was the observation that several of the plasmid injected fish at week 4 and 5 were PCR positive for the 2.9kb amplicon documenting recombination (Fig. 2a) while at the same time, we had difficulties with growing any virus from internal organs (fish 4w-5, 5w-4, 5w-7, and 5w-10; Fig. 2b). Detailed analyses of 4–6 clones from each fish show that recombination occurred and were homologous resulting in the complete 2.9 kb. However, imprecise (homologous) recombination generated either during initial rounds of transcription or during virus replication resulting in deletions (Fig. 3b), also occurred. The deletions varied from one nt deleted to more than 2.2 kb missing fragments, and varied in position and number per clone sequenced. Noteworthy, some of the deletions were identical or varied by only a few nucleotides at start and end positions in several clones (Fig. 3b), and interestingly deletion positions were similar to what has previously been reported by us from field outbreaks^17^. The majority of the deletions in the SAV3 genome, including the majority of deletions repeated in several clones, results in frameshift downstream of the deletions and subsequently non-functional proteins and non-viable viruses. None of the clones had deletions similar to the injected FL-∆6K plasmid (i.e. only 6K gene deleted). In addition, all clones contained single synonymous and non-synonymous substitutions, some also resulting in stop codons, evenly spread in the 2.9 kb region.

### RNA deletion variants occur also in virus injected fish

We cloned and sequenced 5 clones targeting the 2.9 kb amplicon from the 5^th^ passage supernatant of virus isolate 3w-2H that was used for challenge (Fig. 2c). No deletions or single nucleotide substitutions were found in any of the clones. Then, and similar to what was done to confirm recombination in the plasmid-injected fish, RNA isolated from heart of 3w-2H challenged fish (Fig. 2c) were cloned and sequenced (2.9 kb amplicon) and deletions reappeared. 3 to 5 amplicon clones from 3 individuals sampled at 4wpi were sequenced. Of the 13 clones, deletions were found in 4 (Supplementary Fig. 2) and one clone had nucleotide substitution in two adjacent nts, resulting in one amino acid change. This shows that SAV3 accumulates mutations/deletions in experimental *in vivo* challenges at early time post infection.

### Deletions are frequently positioned coincident with predicted RNA secondary structures

**Figure 4:**
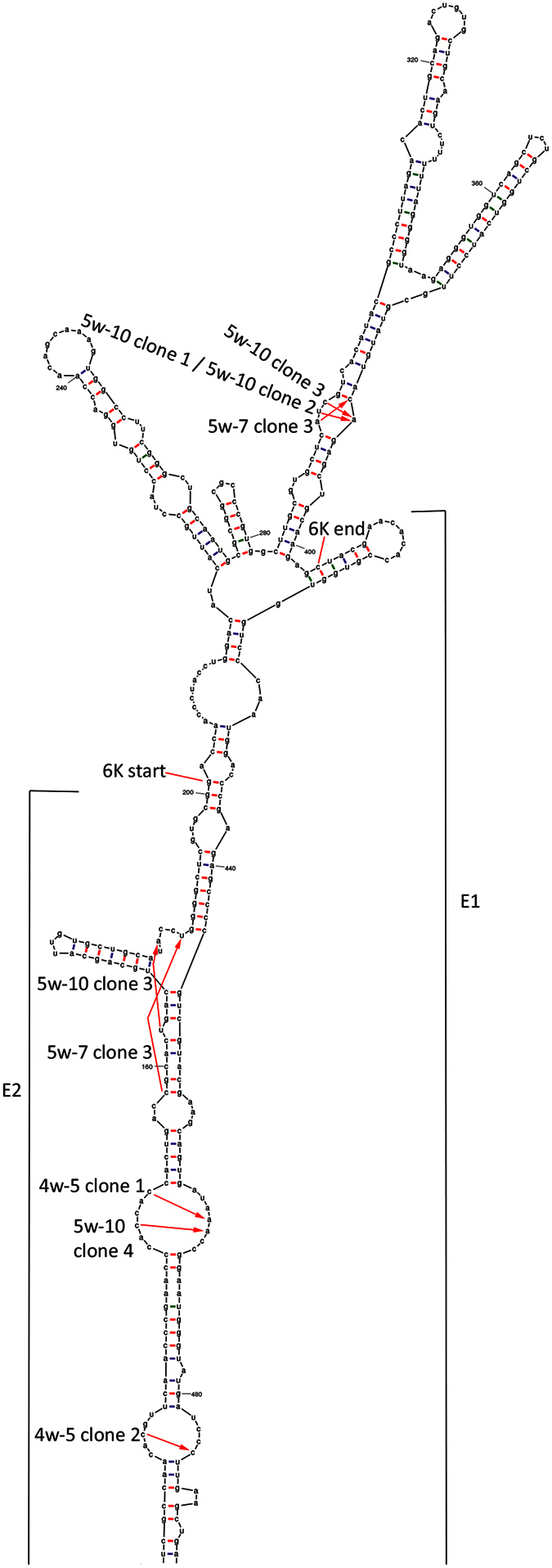
Predicted secondary structure of 6K and surrounding RNA. Example of a predicted secondary structure of RNA with positions indicated by arrows where deletions are initiated and the transcription of RNA continues (Sequence alignment of the sequenced clones with deletions is shown in Supplementary Fig. 3). A sequence covering 200bp in 3′end of E2 gene, 6K gene and 200bp in 5′ end of E1 was submitted for predictions of RNA secondary structure by Mfold Web Server (version 2.3 energies) with folding temperature at 15 °C. The example resulting in highest free energy (ΔG) is given.

In an attempt to shed light on mechanisms possibly involved in generation of deletions, we used Mfold web server^22^ to predict the RNA structure in the region of 6K gene and surrounding nts, since this represents a part of the genome where deletions frequently occur. All deletions found in this region (Fig. 3b) represent defined secondary structures on the predicted example, *i.e*. one single or a larger set of stems of helices (Fig. 4). The start and end nucleotide are related to loops of unpaired nts.

## Discussion

Here we present the first documentation of experimental alphavirus RNA recombination in an animal model. An infectious and virulent virus originated from two i.m. injected cDNA plasmid vectors, each unable to generate infectious progeny alone. Recombination occurred *in vivo* in Atlantic salmon and the resulting virus was shown to be infectious in cell culture. The isolated virus was infectious and resulted in specific pathology in the target organs, *i.e*. heart and pancreas. The recombination is shown to be imprecise as several viral RNA deletion mutants were found after plasmid injection. The deletions were of varying size and genome positions but the location in viral genome was identical or similar for several sequenced clones, and some positions were identical to deletions previously seen in SAV3 RNA in field infections in salmon^17^.

RNA recombination as an evolutionary force in alphavirus is already documented when WEEV and the WEEV antigenic complex descended from a recombination event between a Sindbis-like virus and EEEV^1^,^13^. Thus, recombination seems to be a founding feature of alphaviruses. Still, it has previously not been documented to occur in experimental studies *in vivo* for any Alphavirus species, including marine Alphaviruses. At a general level, studies of RNA virus recombination show that the RNA secondary structure might play a role. This was suggested from the observation that the RNA-dependent RNA polymerases pauses at regions of strong secondary structure resulting in release of the RNA polymerase and the nascent strand from the template^23^,^24^,^25^. Some regions of the SAV3 genome aggregate deletions, as shown here where deletions in and covering 6K gene are highly frequent, also supported by previous reports for variant SAV subtypes^3^,^6^,^17^. The example of Mfold secondary structure modelling given here is indicative of a relation between secondary structures and deletions in and around the 6K gene and this was supported by studies of other structure examples resulting from the Mfold predictions, also including modelling on other selections (length and positions) in the RNA sequence. The indicated relation could be explained by a template-switching incident where the polymerase jumps across or between loops of unpaired nts, *i.e*. deleting stems of helices of base paired nts. Interesting to note is that amplicon sequences from fish tissue revealed a deletion pattern partly similar to what we published from fish samples from field outbreaks^17^. Specific deletions found in the present study, after injection of plasmids or live virus, are also represented in data from 2013, although the size of the deletions could vary with a few nts. In total, the aggregation of deletions in certain “hot spots” of the viral genome is confirmed and secondary structures can be one of several possible underlying mechanisms involved. Recombinant genomes resulting from non-random locations of template switching or from other mechanisms resulting in random locations might be used as templates in several rounds of replication, influenced by selective mechanisms and thereby result in “hot spots”. Non-replicative RNA recombination mediated by RNA secondary structure can also explain the location of the deletions^26^,^27^. In general, future work should include more research on basis and mechanisms for the RNA recombination shown for SAV3, including the putative impact of RNA secondary structures using experimentally confirmed knowledge of the secondary structures.

Some regions of the genome are more prone to be deleted than others while the size, position and frequency in each amplicon varied. In fact, when the 2.9 kb amplicon was produced to confirm recombination, a “ladder” of several shorter products were often seen by gel electrophoresis (Supplementary Fig. 4). These shorter products were not sequenced, but it is reasonable to believe that they represent specific amplicons either resulting from a large deletion or multiple smaller deletions resulting in a short amplicon. Similarly, large deletions in the 2.9 kb amplicon region resulting in shorter amplicons were seen previously when viral RNA recombination was studied *in vitro*^18^. Further, amplicons with large deletions came up from sequencing when approximately 2.9 kb amplicons were cut out from the gel, seemingly as contaminants of smaller PCR amplicons. This could be explained by the PCR enriching shorter amplicons present at high concentration relatively to the 2.9 kb amplicons and a low separation resolution on the gel runs, resulting in low precision. Generation of large deletions fits with the putative mechanisms discussed above. Still, “secondary” deletions in an already deleted RNA copy may result in the deleted fragment increasing in length with repeated rounds of replication.

Alphavirus RNA recombination has earlier been confirmed in experimental *in vitro* studies using RNA transcripts^12^,^14^. Deletion variants of similar characteristics as found here, and suggested to be caused by RNA recombination, have been shown in other alphavirus both *in vivo* and *in vitro* with no presence of cDNA variants of the viral genomes^28^. In addition, equal deletions as presented here were found in SAV3 RNA under field conditions^17^. In total, this supports that the recombination shown in the present work is related to RNA and not the injected DNA plasmids.

Unlike other alphaviruses, SAV is transferred without a vector, which makes the potential host range larger. SAV variants do not only infect salmonids, and while the wild reservoir of SAV remains elusive, SAV subtype 5 have been found in several wild caught fish species in the Shetland Islands and north-east coast of Scotland^29^. Transfer through a vector might restrict genetic variation (bottlenecks) and single amino acids mutations have been associated with overcoming host range barriers especially at the level of the vector^30^,^31^. At the same time as spreading through an insect vector might restrict genetic variation of the virus, the vector serves as a virus pool. For SAV a complicating factor is that Atlantic salmon is a migrating species, which will provide the virus with a paucity of hosts available for infection (under natural conditions in the wild). Thus, it is tempting to speculate that high mutation/recombination rate can provide a genetic plasticity that will benefit the virus in terms of cross-species transmission, also discussed for other Alphaviruses like Chikungunya virus and Venezuelan equine encephalitis virus (VEEV)^30^. A recent study has shown that different variants of SAV isolated from farmed fish in Norway, Scotland and Ireland, represent separate introductions from wild fish species, yet to be identified^32^. The referred study shows that introduction to farmed salmon species very likely occurred as separate events in areas of close proximity. These findings might argue for a viral genome of high plasticity and it has been speculated that the ancestral Alphavirus originates from the marine environment^33^. To achieve a transfer to terrestrial animal species a viral ancestor with a wide host spectre and high genomic plasticity would be beneficial.

Against the ideas above stands a theory that deletions are generated as a by-product of replication and are of no advantage to the viral population. In addition, recombination in RNA viruses is not a result of natural selection that in itself creates an advantageous genotype, but rather an effect of a low-fidelity polymerase and high replication rates producing occasional beneficial combinations^28^,^34^,^35^. Recombination is also linked to the production of defective interfering particles (with similar truncated viral genomes as seen here) which propagates and accumulates at high MOIs and attenuates the virus^14^,^25^,^36^. Our findings do not corroborate this. Although we were not able to sequence more than one clone of the 2.9 kb amplicon from the fish 3w-2 tissue, the gel electrophoresis after PCR show a ladder of smaller amplicons representing sequences with one or several deleted regions of large size (Supplementary Fig. 3). Sequencing of the five PCR clones of the 2.9kb amplicon after the 3w-2H isolate was passaged five times in cell culture show no deletions, no laddering was observed on the gel. We previously found that while deletion mutants are present at high concentrations during natural infections, passage in cell culture tends to result in decrease of deleted RNAs^17^. This would indicate a purifying selection with passage rather than formation of accumulating DI variants. DI particles are well known among Alphaviruses and similar deletion mutants as shown here in the 6K gene are also described previously as non-interfering in VEEV^28^.

An interesting observation here is the low viral load shown by real-time PCR in the 3w-2 fish from which virus was grown. In contrast, we did not succeed in growing any virus from e.g. heart tissue of fish 4w-5 where the real-time PCR indicated approximately 1000 times higher concentration of viral RNA (Fig. 2a,b). These finding corroborate previous studies where primary isolation of SAV from diseased/infected fish has been challenging^17^,^37^,^38^,^39^. For many years, the understanding has been that SAV3 in Norway isolated from farmed salmon is genetically homogenous with little sequence divergence or variability, which is confirmed when comparing sequences of cultivable virus isolates. The observed difficulties isolating virus from tissue might be due to a high concentration of defective RNA, which could indicate that presence of deletion mutants is of some relevance to replication and packaging/release of the virus. More studies dedicated to the prevalence, characteristics and impact of these deletions are needed.

In summary, we have confirmed viral RNA recombination *in vivo*, using the SAV3 subtype of SPDV. This is the first experimental documentation of *in vivo* recombination in *Alphavirus* genus resulting in an infectious virus.

## Material and methods

### Virus and cells

The study was performed using genomic sequences for salmonid alphavirus subtype 3 (SAV3) H10 isolate (GenBank ref. no. JQ799139)^40^, a subtype of salmonid pancreas disease virus (SPDV).

Chum salmon (*Oncorhynchus keta*) heart cells (CHH-1) were used to grow the virus and were obtained from the European Collection of Cell Cultures (ECACC), maintained at 20 °C in Leibovitz’s L-15 media with GlutaMAX^TM^ (Invitrogen), supplemented with 5% foetal bovine serum (FBS) (Sigma Aldrich) and 50 μg/ml gentamicin.

### Model animal

Atlantic salmon parr (*Salmo sala*r L.) for both experimental *in vivo* studies were obtained from the Norwegian institute for water research (NIVA), Solbergstrand, Norway. The average individual weight was 50 g in the first and 28 g in second *in viv*o study. Both studies were conducted in fresh water (city water) at the aquarium facilities of The Norwegian University of Life Science/Norwegian Veterinary Institute in Oslo, Norway. In the first *in vivo* study, the water temperature was 12 ± 1 °C, while in the second study the water temperature was 7 °C the first two weeks and 10 ± 1 °C the remaining four weeks. Six randomly selected fish used in the first study, were tested for persistent infection with infectious pancreatic necrosis virus (IPNV) by PCR and low levels of viral RNA was detected in two. Fish were negative for salmon pancreas disease virus (n = 10). Fish were anaesthetized with 30–40 mg/litre benzocaine prior to handling, *i.e*. injection or sampling. Fish were euthanized by a sharp blow to the head after being put in deep anaesthesia. All experiments were approved by The Norwegian Animal Research Authority and the local IACUC of the Norwegian University of Life Sciences and carried out in compliance with national regulation for the use of animals in experimental research.

### SAV3 6K-deleted cDNA plasmid and helper cDNA plasmid

Construction of the SAV3 6K-deleted cDNA plasmid (FL-∆6K) and the helper cDNA plasmid (Helper-6K) are described in Guo *et al*.^18^. In brief, a full-length infectious clone of SAV3 cDNA was inserted into the pTurboFP635-N vector (Evrogen). Between the vector’s CMV promoter and the 5′UTR region of the full-length genome cDNA, a T7 promoter and a hammerhead (HH) self-cleavage ribozyme sequence was included. The plasmid was designated pSAV3-FL and used subsequently as a template for generation of SAV3 6K-deleted cDNA and helper cDNA plasmids. The 6K gene was deleted by PCR using primers defining the deletion, followed by circularization of the amplified product, resulting in FL-∆6K plasmid. The SAV3 helper 6K cDNA plasmid was constructed by deleting the replicon genes nsP1, nsP2, nsP3 and almost entire nsP4 from the pSAV3-FL, resulting in 100 nt at nsP4 3′-end and intact internal untranslated regions and genes encoding the structural proteins plus 6K, termed Helper-6K (Fig. 1). The plasmids were copied in and purified from transformed competent OneShot TOP10 bacterial cells (Invitrogen), using QIAGEN^®^Plasmid Maxi kit.

### SAV3 FL-∆6K cDNA plasmid and Helper-6K cDNA vector injected in Atlantic salmon parr

Atlantic salmon parr were used in the study. 50 individuals were randomly selected for injection with a plasmid mix of FL-∆6K and Helper-6K and 50 were selected for injection with FL-∆6K only. The fish were injected intramuscularly with 20 μg of each plasmid (40 μg total) diluted in dH_2_O in a total of 100 μl for the FL-∆6K/Helper-6K group, or 20 μg/fish for FL-∆6K group (100 μl injection volume). Fish from the two groups were kept in separate tanks and a third tank with 10 non-injected parr was included as negative control (dH_2_O injected). Ten fish per experimental tank were sampled once a week for five weeks post injection (wpi). At 4 wpi, the number of fish sampled from the two experimental tanks was reduced to 6 and 7, due to mortality experienced between 3 and 4 wpi. The mortality was caused by a *Saprolegnia* infection, commonly seen in recirculating systems and fish handled/stressed. The infection was treated with malachite green (10 mg/L). From the dH_2_O -injected fish in the control tank no mortalities occurred and five individuals were sampled at each time point, 0 weeks and 5 weeks after onset of the experiment (n = 10 total).

### Sampling and sample preservation and preparation

Heart and head kidney tissue were collected and preserved in RNAlater^®^ (Invitrogen). Parallel samples of heart and head kidney were preserved on transport medium (Leibovitz’s L-15 supplemented with 100 mg/ml gentamicin and 30% glycerol). Samples from heart and pancreas were submerged in 10% phosphate-buffered formalin, embedded in paraffin and stained with haematoxylin and eosin using standard methods. Histopathological changes in heart and pancreas was evaluated as described^4^.

### RNA isolation, transcription and PCR based methodology

RNA was extracted from heart and kidney tissue using the RNeasy^®^ Fibrous tissue mini kit on heart specimens and RNeasy^®^mini kit (Qiagen) on kidney, all according to the kit protocols. On-column DNAse treatment was included for all samples. The tissue was homogenized in kit RLT buffer with β-mercaptoethanol using steel beads in a FastPrep-24 homogenizer (MP Biomedicals). RNA was extracted from cell supernatants using QIAamp Viral RNA mini kit (Qiagen) according to the protocol of the manufacturer. RNA was quantified using the Nanodrop ND-1000 spectrophotometer (NanoDrop Technologies). RNA was denatured at 95 °C for 2.5 minutes and transferred directly to ice immediately before cDNA synthesis was performed with SuperScript^®^ III First-Strand Synthesis SuperMix for qRT-PCR (Thermo Fisher Scientific), according to the protocol, using 500 ng RNA in the reaction for heart and kidney samples. For a few samples with RNA concentration too low to include 500 ng, maximum volume of RNA possible was used. Real-time PCR analysis was conducted with Platinum SYBR Green qPCR SuperMix UDG (Thermo Fischer Scientific) as earlier described^17^ using 2 μl 1:2 diluted cDNA as template in a LightCycler^®^ 96 Real-time PCR system (Roche), with primers targeting E2 gene. A conventional PCR was also used as an alternative to real-time PCR, where Q5^®^ High-Fidelity DNA Polymerase (New England BioLabs) was used in combination with primers E1- Fwd and E1- Rev (Supplementary Table) defining a 225 bp product, and run for 35 cycles. A PCR confirming presence of the full 6K gene was also performed using primers 6K Fwd and Insert rev (Supplementary Table) and run for 35 cycles. The resulting 225 and 380 bp product was visualized by gel electrophoresis.

### PCR and sequencing of amplicons

Recombination of virus specific RNA was verified by PCR amplification of a product using a forward primer specific for sequences unique to SAV3 FL-∆6K encoded RNA, Insert Fwd, and a reverse primer resulting in inclusion of unique sequences of Helper-6K encoded RNA, Insert Rev (Supplementary Table). The resulting product after a successful and precise recombination would be sized 2.9 kb and include 67bp of nsP4 (nt 7622–7688 in genome) unique to FL-∆6K in the 5′ end and full 6K gene unique to Helper-6K at the 3′ end, but products of any size, which still include parts of the unique sequences would also indicate recombination. The PCR was run using DyNAzyme EXT DNA Polymerase (Thermo Scientific^TM^) or Q5^®^ High-Fidelity DNA Polymerase for 35 cycles. The products were visualized by gel electrophoresis and products of approximately 2.9 kb were excised and purified with QIAquick gel extraction kit (Qiagen). PCR products were ligated into the pCR 2.1 vector using TOPO TA cloning kit (Invitrogen) before subsequently being transformed into competent OneShot TOP10 bacterial cells (Invitrogen), after manufacturer’s procedures. The insert of purified plasmids of one to six clones was sequenced by commercial services (GATC biotech) using standard vector primers and sequencing primers (Supplementary Table). Full genome sequencing was performed by PCR amplification using four primer sets (Supplementary table) defining overlapping fragments covering the complete genome excluding 5′ and 3′ end sequences and the amplified PCR products (around 3 kb for each fragment) were purified and sequenced (GATC biotech) without subsequent cloning.

### Isolation of recombinant virus from fish tissue

Tissues were homogenized in L-15 medium supplemented with 100 μg/mL gentamicin, centrifuged and obtained supernatant was diluted 1:10 in L-15 (2% FBS and 50 μg/ml gentamicin). IPNV was neutralized by adding K262, a custom made rabbit serum to IPNV^41^ to the tissue homogenate, and incubated for one hour at room temperature. Heart tissue supernatant (from homogenate) was further diluted 1:10 and 1:100, and kidney 1:50 and 1:100. 24 well plates with 50% confluent CHH-1 cells was inoculated with 250 μl per well of the different dilutions. The inoculated cells were propagated at 15 °C until the 3^rd^ passage with the following passages at 10 °C. At passage, virus supernatant was harvested by one freeze-thaw cycle (passage 1–3) at 14dpi unless cytopathic effect (CPE) was observed (late passages). Supernatant was clarified by centrifugation before transferring to a naïve cell culture. Anti-IPNV antiserum (K262) was added to the inoculum up to 4^th^ passage, and real-time PCR confirmed all supernatants IPNV negative. IPNV real-time PCR was performed as described above using specific IPNV primers (Supplementary table).

SAV3 titre of the 5^th^ and final supernatant were determined by endpoint dilution on CHH-1 cells grown in 96-well plates, estimating the TCID_50_ by the method of Kärber^42^. Detection of SAV3 viral proteins in cultured CHH-1 cells was performed by IF assay on the 2^nd^ passage using a polyclonal antibody against SAV3 structural proteins^18^.

### Recombinant SAV3 *in vivo* challenge

A challenge study was conducted using 22 Atlantic salmon parr. In the experimental tank, 12 fish were injected intramuscularly with 100 μl 10^6.8^ TCID_50_ of recombined SAV3 (3w-2H isolate) from the 5^th^ cell culture passage. 10 salmon parr were injected with 100 μl PBS, kept in a separate tank and used as negative controls. Six fish were sampled from the challenge tank at 4 and 6 wpi. Five fish from the negative control tank were sampled at 0 and 6 wpi. Sampling was performed and the samples preserved and analysed as described above. Presence of SAV3 antigens *in situ* in heart and pancreas was documented by immunohistochemistry^21^.

## Additional Information

**How to cite this article**: Petterson, E. *et al*. Experimental piscine alphavirus RNA recombination *in vivo* yields both viable virus and defective viral RNA. *Sci. Rep*. **6**, 36317; doi: 10.1038/srep36317 (2016).

**Publisher’s note:** Springer Nature remains neutral with regard to jurisdictional claims in published maps and institutional affiliations.

## Supplementary Material

Supplementary Information
