# Supplementary material for: Experimental piscine alphavirus RNA recombination in vivo yields both viable virus and defective viral RNA
Source: Sci Rep. 2016 Nov 2;6:36317. doi: 10.1038/srep36317 (PMC5090867; doi:10.1038/srep36317)
Supplement: Supplementary Information [file srep36317-s1.pdf]

a)

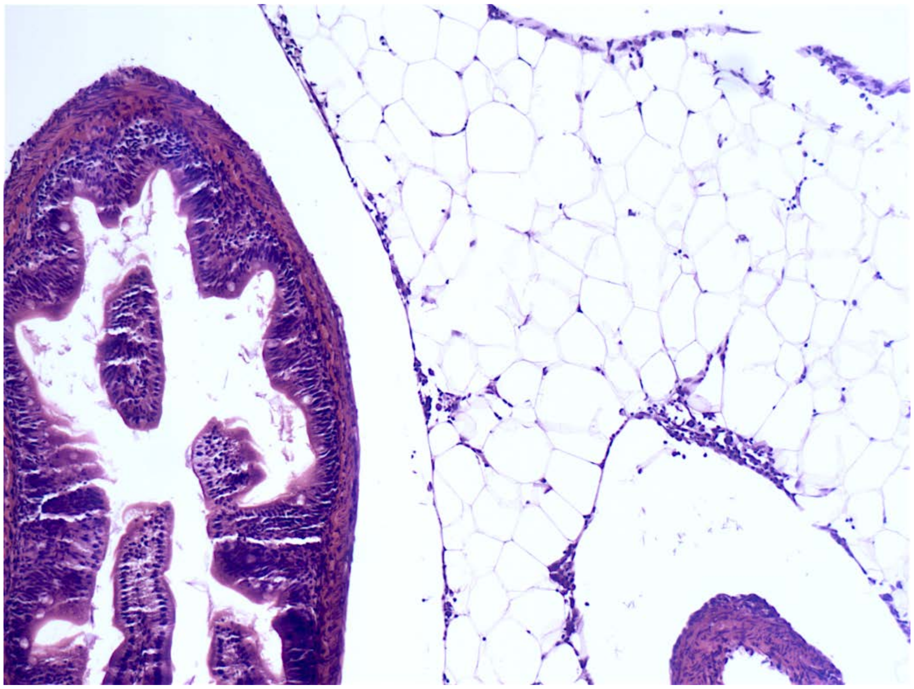

b)

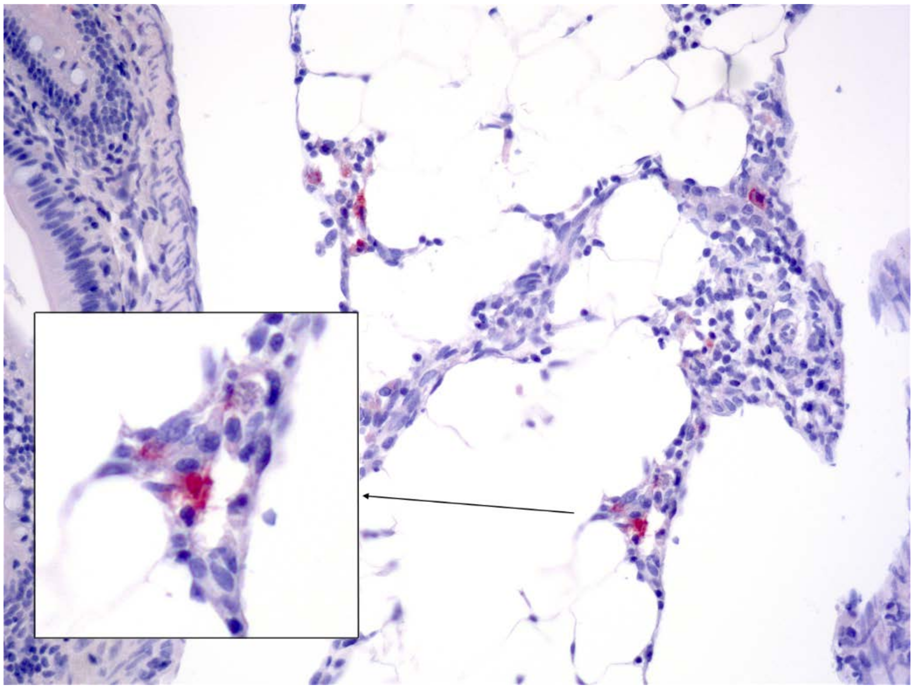

Supplementary Figure 1 | Pathological changes caused by recombined SAV3 in Atlantic salmon.

Histological tissue preparations of pancreas from Atlantic salmon injected with recombined SAV3, 3w-2H isolate, at 4 wpi. **a**) Loss of exocrine pancreas in perivisceral fat tissue. **b**) Immunohistochemistry of exocrine pancreatic tissue. Red coloration indicates presence of SAV3 antigen.

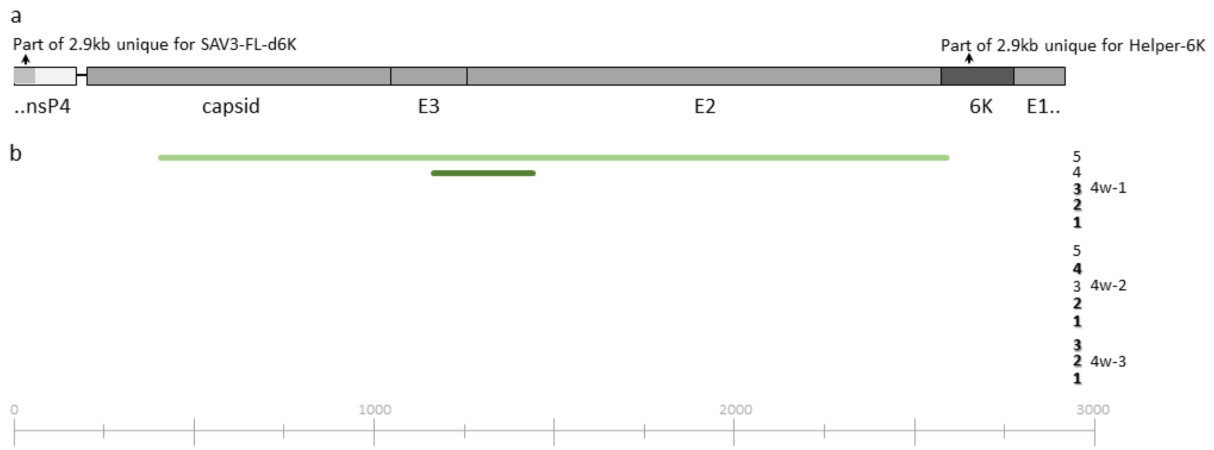

Supplementary Figure 2 |Sequencing of PCR products from recombined SAV3 injected fish.

a) Overview of full-length 2.9kb PCR product including sequences unique for SAV3 FL-Δ6K and Helper-6K

b) Deletions per single PCR product clone relative to full-length 2.9kb product are marked as lines, dots represents deletions of <10nt.

Colour-coding has been used to visualize fish individuals and colour shades differentiating each clone, also numbered with digits. Clone numbers in bold is used on clones with no deletions.

Supplementary Figure 3 |Sequence alignments.

```
FL         TCGCCAACACGTTCAACCCGAACCCACCACCACTGACCGCACTGACTGCAGCATTGTGCTGCATACCTGGGGCTCGTGCGGACCAACCCTACCTGGACAT
5w7-3      TCGCCAACACGTTCAACCCGAACCCACCACCACTGACC-----------------------------TGGGGCTCGTGCGGACCAACCCTACCTGGACAT
5w10-1     TCGCCAACACGTTCAACCCGAACCCACCACCACTGACCGCACTGACTGCAGCATTGCGCTGCATACCTGGGGCTCGTGCGGACCAACCCTACCTGGACAT
5w10-2     TCGCCAACACGTTCAACCCGAACCCACCACCACTGACCGCACTGACTGCAGCATTGTGCTGCATACCTGGGGCTCGTGCGGACCAACCCTACCTGGACAT
5w10-3     TCGCCAACACGTTCAACCCGAACCCACCACCACTGACCGCACT---------------------ACCTGGGGCTCGTGCGGACCAACCCTACCTGGACAT
4w5-1      TCGCCAACACGTTCAACCCGAACCCACCA-----------------------------------------------------------------------
5w10-4     TCGCCAACACGTTCAACCCGAACCCA--------------------------------------------------------------------------
4w5-2      TCGCCAACA-------------------------------------------------------------------------------------------


FL         CATTGCCTACCTGTGGACCAACAGCAAAGTGGCCTTCGGGCTGCAATGCGCGGCGCCCGTGGCTTGCGTGCTCATCGTCACATACGCCCTTAGACACTGC
5w7-3      CATTGCCTACCTGTGGACCAACAGCAAAGTGGCCTTCGGGCTGCAATGCGCGGCGCCCGTGGCTTGCGTGCTCA--------------------------
5w10-1     CATTGCCTACCTGTGGACCAACAGCAAAGTGGCCTTCGGGCTGCAATGCGCGGCGCCCGTGGCTTGCGTGCTCAT-------------------------
5w10-2     CATTGCCTACCTGTGGACCAACAGTAAAGTGGCCTTCGGGCTGCAATGCGCGGCGCCCGTGGCTTGCGTGCTCAT-------------------------
5w10-3     CATTGCCTACCTGTGGACCAACAGCAAAGTGGCCTTCGGGCTGCAATGCGCGGCGCCCGTGGCTTGCGTGCTCATC------------------------
4w5-1      ----------------------------------------------------------------------------------------------------
5w10-4     ----------------------------------------------------------------------------------------------------
4w5-2      ----------------------------------------------------------------------------------------------------


FL         AGACTGTGCTGCAAGTCTTTTTTAGGGGTAAGAGGGTGGTCAGCTCTGCTGGTCATCCTTGCGTATGTACAGAGCTGCAAGAGCTACGAACACACCGTGG
5w7-3      ---------------------------------------------------------------------CAGAGCTGCAAGAGCTACGAACACACCGTGG
5w10-1     ----------------------------------------------------------------------AGAGCTGCAAGAGCTACGAACACACCGTGG
5w10-2     ----------------------------------------------------------------------AGGGCTGCAAGAGCTACGAACACACCGTGG
5w10-3     ----------------------------------------------------------------------AGAGCTGCAAGAGCTACGAACACACCGTGG
4w5-1      ----------------------------------------------------------------------------------------------------
5w10-4     ----------------------------------------------------------------------------------------------------
4w5-2      ----------------------------------------------------------------------------------------------------


FL         TGGTCCCAATGGACCCGAGAGCCCCGTCGTACGAAGCAGTGATAAACCGGAATGGGTATGATCCCTTGAAGCTGA
5w7-3      TGGTCCCAATGGACCCGAGAGCCCCGTCGTAC-AAGCAGTGATAAACCGGAATGGGTATGATCCCTTGAAGCTGA
5w10-1     TGGTCCCAATGGACCCGAGAGCCCCGTCGTACGAAGCAGTGATAAACCGGAATGGGTATGATCCCTTGAAGCTGA
5w10-2     TGGTCCCAATGGACCCGAGAGCCCCGTCGTACGAAGCAGTGATAAACCGGAATGGGTATGATCCCTTGAAGCTGA
5w10-3     TGGTCCCAATGGACCCGAGAGCCCCGTCGTACGAAGCAGTGATAAACCGGAATGGGTATGATCCCTTGAAGCTGA
4w5-1      ---------------------------------------------ACCGGAATGGGTATGATCCCTTGAAGCTGA
5w10-4     --------------------------------------------AACCGGAATGGGTATGATCCCTTGAAGCTGA
4w5-2      -----------------------------------------------------------------TTGAAGCTGA
```

Alignments of example sequences from clones of 2.9kb amplicon showing deletions in 6K and surrounding RNA presented with predicted secondary structure in Fig 4. FL – full length sequence.

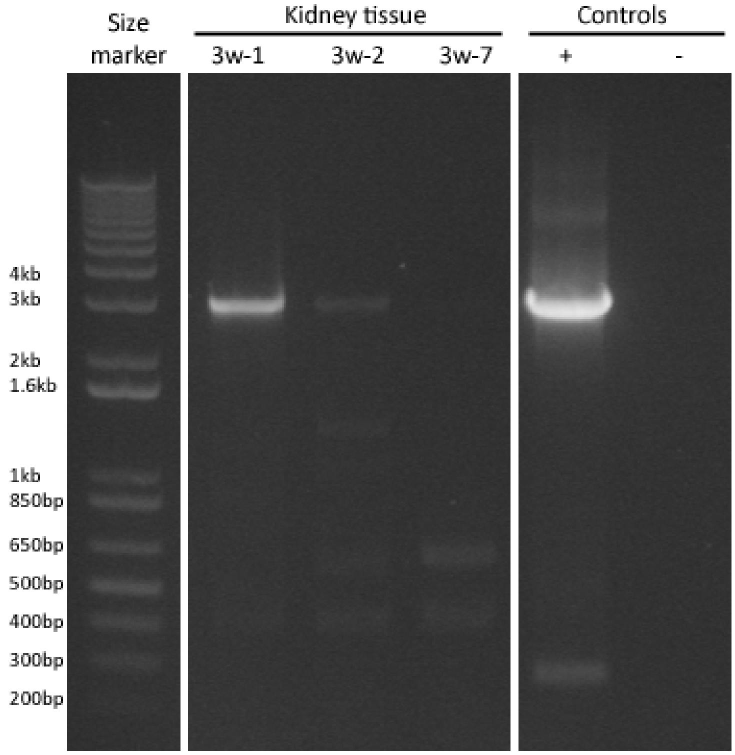

Supplementary Figure 4 | Deletions illustrated as a ladder of PCR products visualized by gel electrophoresis

PCR products amplified from primers for 2.9kb amplicon were visualized on an agarose gel for individual 3w-1, 3w-2 and 3w-7 kidney tissue. Several shorter products than the expected 2.9kb size are shown for 3w-2 and 3w-7.

Experimental piscine alphavirus RNA recombination *in vivo* yields both viable virus and defective viral RNA
Elin Petterson, Tz-Chun Guo, Øystein Evensen and Aase B. Mikalsen

Supplementary information

Supplementary table | Primers used for PCR reactions and genome sequencing

**Primers used for PCR reactions**	**Primer sequence (5´to 3´)**	**Annealing**
IPNV For	TGGCATTCTTGTTTGTTTCCT	58°C
IPNV Rev	CGTCCCGTTCAGAGCATAGA	
Ins For	AAGTGGAAAGCTGGTACAGAGTGGG	59°C
Ins Rev	GAGCTGTAGTTGGTGAGATGACG	
6K For	GAGCTGTAGTTGGTGAGATGACG	58°C
E1 For	TATCCGGATTCTTTTAGAGC	59°C
E1 Rev	GTAGAGATCTGTTCGGCAAT	
**Primers used for sequencing of 2.9kb**		
Ins For	See above	
S2	ACATCAAGTTCCAGGTCGCCGA	
S3	GTTCTCAATCGGGAGTGACCGCTAA	
S4	TGACCGCACTGACTGCAG	
Ins Rev	See above	
**Primers used for full genome sequencing of 5th passage**		
F1-For *	CCGCCGGCACTACAGTCACTGTA	60°C
F1-Rev*	ACTGCCCAACAGGTGTTACGCTTC	
F1-1	ACAAGCTGAAAAGCTGGCACCTG	
F1-2	AACGTCAACTCCATGGAT	
F1-3	GTGTTGCGCACCAGGAAA	
F1-4	AAAATAGCCGCACGTACG	
F2-For *	ATAATGAGCTCATGACTGCGGCTGC	60°C
F2-Rev *	CTCTTGTTCTTTTGAGTGGGGCGC	
F2-1	CCGCTGATCCCTATACTC	
F2-2	CAGTTGACAGATGGGACC	
F2-3	CCCTCAACTGACCGACAT	
	AAGAGGAAGAGGAACATG	
F2-5	GCACAGCCACGAGCCGCC	
F3-For *	GCCTGCAGAGACGAAGAAGAAGCT	60°C
F3-Rev*	GTCCCTTCCCAGGTATCGCAGAG	
F3-1	ATGGCCGCCACCAAGCGC	
F3-2	GAAGACCTCCTGACCCTC	
F3-3	CGCGCGAGGGGAGTACGT	
F3-4	GCCGAGGACATGGACCTC	
F4-For *	TACGACACACAAATCCTCGCCG	60°C
F4-Rev*	GCGGCACTTCTTCACCACGCAGT	
F4-1	CCGTGCTGCTGACTACACGGA	
F4-2	GTGGCTGCCACCGTCTCT	

* Primers also used for PCR amplification of fragment 1-4.
